# Infectious Bronchitis Fibrinosa in Cattle*Wochenschrift für Thierheilkunde, No. 19, 1884.

**Published:** 1884-10

**Authors:** 


INFECTIOUS BRONCHITIS FIBRINOSA IN CATTLE.*—District Veterinarian Mayrwieser reported quite a number of cases of this rare complication in cattle at a late meeting of the Munich Veterinary Association. (He calls it "infectious bronchial croup," but as the word "croup" has only reference to the clinical phenomena, by which we designate the peculiar manner of breathing, and as the exudation in such cases is of a decidedly fibrinous nature, it is much better to designate such occurrences from the pathological rather than the clinical standpoint.)

In a certain stable there were 50 oxen for fattening, 32 of which had been lately placed there. These two lots had been purchased in different localities. Among them was a 6-year-old that was troubled with a short, dry, croupy cough. On examination, an unimportant tuberculous infiltration-pneumonia was discovered occupying the right lung. On the 12th of the following month it was reported that this ox was greatly troubled in its respiration; also a second that stood beside it. The condition of the first ox was then as follows: Head elevated and extended, a copious secretion flowed from the nose,

* Wochenschrift für Thierheilkunde, No. 19, 1884.

respiration 50 and difficult, stertorous, muffle dry, the pneumonic centre had increased in dimensions, bronchial respiration in these parts distinct, increased vesicular respiration in other portions of lung, temperature, 40.8 deg , C., pulse weak and wiry, 66 ; it was singular that the pulse did not bear any correspondence to the severity of the complication.

The animal was slaughtered with the consent of the owner as no accurate diagnosis of the disease could be made.

Autopsy. Contents of abdominal cavity normal. Pneumonia conformable to clinical diagnosis. Serous infiltration of interstitial tissue of lungs, bronchi filled with fibrinous exudation which was firmly attached to their walls. This condition was far more extensive in the trachea. Under the exudated pseudo-membrane the mucosa was red—hyperæmic ; the pseudo-membrane in these parts was easily detached, and in many places had become so intra, without leaving a wounded and bleeding surface. This condition extended to the larynx. The mucosa of the nasal cavities and pharynx was greatly swollen and covered by a soft, thin, pseudo-membrane.

As already mentioned, on the 12th of November a second ox had been attacked by a similar complication ; from this time the disease extended to the others, two being attacked on the 13th, two more oxen and a calf on the 14th, four oxen and a cow on the 15th, and still another ox on the 16th. The disease did not extend further and all finally recovered. The phenomena were the same in each case.

